# TRPV1: A Target for Next Generation Analgesics

**DOI:** 10.2174/157015908784533888

**Published:** 2008-06

**Authors:** Louis S Premkumar, Parul Sikand

**Affiliations:** Department of Pharmacology, Southern Illinois University School of Medicine Springfield, IL 62702, USA

**Keywords:** TRPV1, nociceptors, protein kinases, inflammatory mediators, pain, nociceptive ion channels.

## Abstract

Transient Receptor Potential Vanilloid 1 (TRPV1) is a Ca^2+^ permeant non-selective cation channel expressed in a subpopulation of primary afferent neurons. TRPV1 is activated by physical and chemical stimuli. It is critical for the detection of nociceptive and thermal inflammatory pain as revealed by the deletion of the TRPV1 gene. TRPV1 is distributed in the peripheral and central terminals of the sensory neurons and plays a role in initiating action potentials at the nerve terminals and modulating neurotransmitter release at the first sensory synapse, respectively. Distribution of TRPV1 in the nerve terminals innervating blood vessels and in parts of the CNS that are not subjected to temperature range that is required to activate TRPV1 suggests a role beyond a noxious thermal sensor. Presently, TRPV1 is being considered as a target for analgesics through evaluation of different antagonists. Here, we will discuss the distribution and the functions of TRPV1, potential use of its agonists and antagonists as analgesics and highlight the functions that are not related to nociceptive transmission that might lead to adverse effects.

## INTRODUCTION

Pain is manifested as nociceptive (noxious stimuli), inflammatory (tissue damage) and neuropathic (neuronal lesion) pain. Noxious stimuli are transduced by peripheral nociceptors, which transmit nociceptive information to pain processing areas in the brain *via* the spinal cord. Sensory nerve endings express chemo-, mechano-, and thermo-sensi-tive ion channels, which include acid sensitive ion channels (ASIC), degenerin/epithelial sodium channels (DEG/ENAC), adenosine triphosphate (ATP) gated ion channels (P2X), and transient receptor potential (TRP) channels [28, 49, 116, 170]. TRP channels (TRPVanilloid, TRPAnkyrin, TRPClassical, and TRPMelastatin) are chemo-, mechano-, and thermo-sensitive [60, 123]. These receptors are sensitized by proinflammatory agents, the receptors of which are coupled to intracellular signaling pathways and mediate heightened pain perception. 

TRPV1 is a transducer of noxious temperature and chemi-cal stimuli [31]. It can initiate nociceptive signaling by generating a receptor potential at the peripheral nerve endings by increasing membrane permeability to monovalent and divalent cations including Ca^2+^. TRPV1 is sensitized by inflammatory mediators and is responsible, in part for inflammatory pain arising from tissue injury [65, 76, 86]. TRPV1 expression is increased in neuropathic pain resulting from nerve lesion [55, 72]. Retrograde transport of nerve growth factor (NGF) released at the site of peripheral tissue injury to the DRG soma results in activation of p38 mitogen-activated protein kinase (p38 MAPK)[76]. Enhanced translation and transport of the TRPV1 protein selectively to the peripheral terminals of sensory neurons is suggested to underlie thermal hypersensitivity following tissue injury [76]. This is generally referred to as peripheral sensitization, although increased expression of TRPV1 at the central terminals of DRG neurons could contribute to central sensitization. We have recently demonstrated that PKC-mediated phosphorylation of TRPV1, expressed on the central terminals of sensory neurons, activates the receptor at body temperature resulting in enhanced glutamatergic synaptic transmission [162]. Increased neuronal activity in primary afferents could augment the activity of second order dorsal horn neurons and third order thalamic neurons. This in turn, may be interpreted as heightened pain by cortical pain sensing areas. The finding that TRPV1 knock-out mice are less susceptible to certain modalities of pain, suggests the possibility of TRPV1 antagonists as the next generation of analgesics. The selectivity of TRPV1 as a target is bolstered by the reports that TRPV1 knock-out or ablation of TRPV1 containing neurons by neonatal administration of capsaicin or resiniferatoxin (RTX) does not exhibit other obvious abnormalities [30, 135, 175]. However, it is yet to be determined how the block of TRPV1 will impact in a preexisting disease state.

## DISTRIBUTION OF TRPV1

### Peripheral Nervous System

A subset of primary sensory neurons with soma in dorsal root ganglia (DRG), trigeminal ganglia (TG) and nodose ganglia expresse TRPV1 [31, 67]. These are peptidergic, small to medium diameter neurons that give rise to un-myeli-nated C-fibers and thinly myelinated Aδ-fibers. TRPV1 is also expressed in neurons that are labeled for-D-galactosyl-binding lectin IB_4_ and express the ionotropic ATP receptor P_2_X_3_ [67, 68]. In inflammatory conditions, capsaicin insensitive neurons can express TRPV1 [8, 9]. Tissue or nerve injury may also change the expression of neurotransmitter receptors and signaling molecules in nociceptors, which may underlie chronic pain conditions [199]. Central terminals of vanilloid-sensitive neurons form synapses on the dorsal horn of the spinal cord (DRG neurons) or the spinal nucleus of the trigeminal tract (TG neurons) [105, 106, 107] transmitting nociceptive information to the CNS. 

TRPV1 expression has been established in non-neuronal cells like mast cells [24], glial cells [23], bronchial epithelial cells [194], uroepithelial cells [21, 22] and keratinocytes [47, 75, 166]. The role of TRPV1 in different regions of the body is briefly discussed below. 

### Central Nervous System

Expression of TRPV1 in various regions of the brain has been successfully established using an array of techniques including RT-PCR, *in situ* hybridization and [^3^H]-RTX binding [2, 31, 99, 100, 119, 147, 172, 184]. Application of capsaicin resulted in an increase in the excitatory glutamatergic synaptic transmission in the nucleus tractus solitarius (NTS) [52], locus coeruleus [111], dopaminergic neurons of the substantia nigra [110] and the preoptic hypothalamic neurons [85]. In these experiments, the facilitatory effects of capsaicin on the transmission are due to activation of presynaptic TRPV1 receptors resulting in enhanced glutamate release. However, acute capsaicin application has also been reported to produce postsynaptic effects in NTS slice preparations [52], suggesting the expression of TRPV1 in second order neurons. TRPV1 activation in cortical slices also results in glutamate release [151]. In addition, iontophoretic application of capsaicin in the periaqueductal gray produced analgesia, which was inhibited by NMDA receptor blockers [132]. Interestingly, in another study, low concentrations (10 nM) of capsaicin resulted in hyperalgesia, as indicated by increased neuronal activity in the ventral medulla and decreased latency of nocifensive behavior [115]. Furthermore, peripheral application of capsaicin enhanced bursting activity in the dorsal raphe nucleus, enhanced firing rate and bursting activity of dopaminergic neurons when applied to ventral tegmental area [109], but inhibited neuronal spike activity in the cerebellum [150]. Activation of medial thalamic neurons by capsaicin enhanced the firing frequency of these neurons, which was inhibited by morphine [11]. 

TRPV1 expression has been demonstrated in the basal ganglia by enhanced locomotion, vasodilation and hypothermia induced by capsaicin injection [43, 44]. The physiological functions of TRPV1 in the hippocampus are unclear [7, 93]. TRPV1 activation by its agonists capsaicin, RTX and anandamide in the CA1 region of hippocampal slices resulted in the facilitation of paired pulse depression of spike potentials [7]. The enhancement of inhibitory synaptic transmission is suggested to result from the influx of Ca^2+^ through TRPV1, expressed on the presynaptic terminals of GABAergic neurons. However, this could not be demonstrated in ex-vivo assays performed to study the effects of TRPV1 activation on K^+^-evoked Ca^2+^ entry and release of radiolabeled GABA from rat hippocampal nerve terminals [7]. Recently, it has been demonstrated that TRPV1 knock-out mice show reduced anxiety-related behavior [112]. These animals also exhibited less freezing following auditory fear conditioning and stress sensitization as compared to their wild type littermates [112]. In addition, the knock-out mice demonstrated deficits in developing long-term potentiation in the Schaffer collateral-commissural pathway stimulation, which may underlie reduced fear and anxiety behavior [112]. 

Intraperitoneal administration of capsaicin and endocannabinoid uptake inhibitor AM404 produced a drop in body temperature. Pre-administration of capsazepine but not cannabinoid receptor antagonist inhibited the hypothermic effects of both capsaicin and AM404, underscoring the importance of TRPV1 in regulating body temperature [142]. Furthermore, elevated body temperature has been reported in capsaicin-desensitized animals as compared to control or TRPV1^-/-^ mice [177]. Capsaicin-desensitized animals have been previously reported to exhibit hyperthermia in response to systemic administration of the E. coli endotoxin, lipopolysaccharide (LPS) [176]. In contrast to these findings, Iida and colleagues [74] reported normal body temperature regulation in TRPV1^-/-^ mice and hypothermia in response to systemic LPS administration, suggesting a role of TRPV1 in temperature regulation at a peripheral site. Recently, an interesting *in vivo* study evaluating the efficacy of various TRPV1 antagonists in clinical development reported that antagonism of the TRPV1 receptor resulted in hyperthermia [56]. Lipophilic TRPV1 antagonists that crossed the blood-brain barrier as well as peripherally acting antagonists induced comparable hyperthermia suggesting that TRPV1 is tonically activated and involved in regulating body temperature at sites mainly in the periphery [56]. Thus, the exact mechanisms involving TRPV1 in body temperature regulation need to be further examined and hence caution should be exercised designing and testing TRPV1 antagonists. 

### Cardiovascular System

The heart is richly innervated by sensory and vagal nerve endings. These nerves transduce chemical and mechanical changes from the heart to the brain. Sensory nerve endings supplying the heart express TRPV1 [202] and are able to release vasoactive peptides like CGRP and SP upon activation. The endogenous activator of TRPV1 in the heart are possibly protons, as lactic acid is produced during ischemia. Lactic acidosis and extracellular acidification are important in inducing depolarization during cardiogenic sympthoexcitatory reflex (CSR) [17, 116, 134, 153, 170]. Ablation of TRPV1-expressing afferent nerve endings by RTX administration has been reported to abolish CSR mediated cardiac pain [202]. Furthermore, reactive oxygen and nitrogen species (ROS and RNS) liberated during myocardial infarction and reperfusion could activate TRPV1 present on the sensory nerve endings [154, 155]. 5-Lipoxygenase (5-LOX) metabolites of arachidonic acid have been shown to activate cardiac sensory afferents possibly by activating TRPV1 during myocardial ischemia [169].

Vasoactive peptides modulate cardiac functions in a complex manner. Capsaicin-induced neuropeptide release may induce vasodilatation, enhance cardiac blood flow and facilitate recovery from ischemia [188]. On the contrary, it has been suggested that generation of arachidonic acid metabolite, 20-hydroxyeicosatetraenoic acid (20-HETE) results in vasoconstriction *via* the activation of TRPV1. Destruction of c-fiber nerve endings by capsaicin, antagonism of TRPV1 using capsazepine, ruthenium red or inhibiting substance P (SP) receptor by its antagonist have been shown to prevent myogenic constriction [156]. 

### Respiratory System

The expression of TRPV1 in the sensory nerve endings arising from the nodose ganglia is well established [119]. Neurogenic inflammation plays an important role in the pathogenesis of diseases, such as asthma and chronic obstructive pulmonary disease. TRPV1 agonists induce bronchoconstriction and airway hyperactivity, enhance mucous secretion and induce microvascular exudation [58, 88]. These effects are mediated in part by SP and calcitonin gene related peptide (CGRP) released from the nerve terminals upon TRPV1 activation. Furthermore, sensitization of TRPV1 by inflammatory mediators like prostaglandins (PGs) [108, 125], bradykinin (BK) [35, 138, 139], nerve growth factor (NGF) [35, 76, 161] and LOX metabolites of arachidonic acid [71, 73] may lead to exacerbation of asthma. Studies have shown that the sensitivity of capsaicin-induced cough reflex/response is heightened in chronic airway inflammatory diseases [58]. TRPV1 is also shown to be involved in citric acid-induced cough [95, 186], gastroeosphageal reflux-induced asthma as well as airway disorders induced following inhalation of acid fog or pollutants [62, 65, 95]. Thus, selective TRPV1 antagonists may prove promising in treating many inflammatory airway disorders.

### Urinary Bladder

TRPV1 in the urinary system is associated with perception of pain and regulating the micturition reflex. Its expression in the urinary bladder, ureter, urethra and renal pelvis of rats has been established by using [^3^H] RTX and immunohistochemistry [1, 13, 21, 99, 135, 174, 182]. TRPV1 has been reported to co-localize with CGRP, and SP receptors in the rat urinary bladder [13, 14, 41].

TRPV1 plays an important role in enhancing bladder reflex contractions in the chronically inflamed bladder [27, 37, 48, 98] and its responses can be significantly potentiated by the activation of PKC [157]. Application of capsazepine resulted in decreased contractions in the bladder inflamed by cyclophosphamide [48]. Furthermore, in acetic acid or lipopolysaccharide (LPS)-induced inflammation of the bladder, the frequency of urinary bladder contractions was significantly enhanced in wild type TRPV1 mice but not in TRPV1 knock-out mice. The frequency of bladder reflex contractions were reported to be similar in both TRPV1^+/+^ and TRPV1^-/-^ mice and it was shown that high concentrations of capsazepine had no effect on the bladder reflex activity of normal bladders [48]. In contrast to these results, Birder and colleagues [21] reported that TRPV1^-/-^ mice exhibited high frequencies of non-voiding contractions, increased bladder volume and enhanced the frequency of low-volume urinations, suggesting that TRPV1 is involved in normal bladder functions. Conflicting data from different groups suggest that TRPV1 plays an important yet complex role in regulating bladder function. 

Intravesical administration of capsaicin and RTX, a potent TRPV1 agonist results in a marked decrease in TRPV1, SP and CGRP immunoreactivity in the rat urinary bladder [13, 14], which may be as a result of nerve terminal degeneration mediated by these agents [27, 35, 37, 40, 146, 163]. This is the underlying mechanism for the successful demonstration of TRPV1 agonists in treating interstitial cystitis [99] and urinary bladder hyperreflexia [37, 45, 46]. In this regard, therapeutic potential of RTX in treating urinary incontinence is superior to capsaicin [97, 98]. Capsaicin administration is associated with rapid depolarization of the pain transmitting c-fibers, which is perceived as a burning pain. However, RTX slowly depolarizes the c-fibers resulting in depolarization block leading to minimal pain during administration [140]. Unfortunately, the high lipophilicity of RTX poses problems with efficient packaging and loss of potency after reconstitution. RTX is in clinical trials for treating urinary bladder hyperreflexia. 

### Gastrointestinal Tract

TRPV1 immunoreactivity has been observed in the visceral sensory afferents and on the vagal afferents that supply the stomach mucosa, musculature and enteric plexuses [10, 136, 152, 165, 197]. Visceral sensory neurons express CGRP and SP [59, 67, 148, 197]. Capsaicin-sensitive neurons in the gut serve both afferent and efferent functions [66, 105]. Transmission of gastric pain sensation to the higher centers of the brain constitutes their afferent function, whereas release of CGRP and SP in the periphery reflects their efferent function. Neurons of nodose ganglion contain high levels of TRPV1 and 70% of the vagal afferents are sensory in nature [120]. 

TRPV1 activation in the foregut by capsaicin produces gastric hyperemia and enhances gastric protection by increasing mucus and HCO_3_^-^ secretion [65, 69, 84, 86, 87, 180, 181]. The protease-activated receptor 2 (PAR2), a Gq-coupled receptor is suggested to sensitize TRPV1 by PKC activation in the stomach, enhancing gastric protection by increasing gastric mucus secretion. This effect is TRPV1 specific as it was inhibited by capsazepine and suggested to occur as a result of CGRP and tachykinin release upon TRPV1 activation [86]. One of the major issues with conventional COX 1 and 2 inhibiting non-steroidal anti-inflammatory agents is their ability to increase gastric acidity, leading to bleeding. However, this effect has been attributed to a lack of PG synthesis and action on their receptors (IP, EP2 and EP3 in the duodenum and EP1 in the stomach) [12, 126, 178], which causes an increase in the acid secretion. PGs do not directly induce nociception, but they are able to sensitize nociceptive ion channels to bring about pain perception. In this context, it is worth noting that the same sensitizing effect could play a role in the gastric mucosa. Application of capsaicin has been shown to increase HCO_3_^-^ secretion as effectively as acid-induced HCO_3_^-^ secretion [6]. This effect could be specifically blocked by ablation of capsaicin sensitive neurons, capsazepine, a TRPV1 antagonist, indomethacin (COX 1 and 2 inhibitors) and N(G)-nitro-L-arginine methylester (L-NAME), a NO synthesis inhibitor. Capsaicin increased mucosal PGI2 but not PGE2 levels, corroborating this observation that only PGI2 receptor knock-out mice abolished capsaicin induced HCO_3_^-^ secretion. Capsaicin-induced HCO_3_^-^ secretion is contrary to the myth that food containing the hot chili pepper can worsen gastric acidity. One would expect an acid-induced effect to overlap that of a capsaicin-induced effect, given the ability of acid to activate TRPV1 potently. Interestingly, capsaicin-induced acid secretion was blocked by capsazepine, L-NAME, indomethacin, in IP receptor knock-out mice and ablation of TRPV1 containing neurons but not by the BK 2 receptor antagonist or the EP1 receptor antagonist [6]. However, the acid induced HCO_3_^-^ secretion was inhibited by indothemacin, L-NAME, EP1 antagonist and sensory deafferentation but not by capsazepine [6]. Thus, acid and capsaicin enhance HCO_3_^-^ secretion through common players but likely through different receptors.

TRPV1 mediated CGRP release could play a role in increasing blood supply to malignancies of the GI tract; the beneficial effect of COX inhibitors in this regard may arise from their ability to reduce TRPV1-mediated CGRP release and inhibition of PG-mediated angiogenesis. TRPV1 is associated with inducing inflammation and tissue damage in the pancreas, ileum and colon following dextran sulphate sodium-induced colitis in rats [90, 91], caerulein-induced pancreatitis [129], *Clostridium difficile* toxin A- and endocannabinoid-induced colitis [117, 118]. These effects of TRPV1 are associated with release of SP from the sensory afferents that activates the neurokinin receptor resulting in an activation of immune cells, mast cells and enteric cells causing hypersecretion, inflammation and finally mucosal damage [65, 117]. Patients diagnosed with active inflammatory bowel disease demonstrate a greatly increased TRPV1 immunoreactivity in colonic nerve fibers [197, 201], suggesting an important implication of the TRPV1 antagonists in treating GI distress. Recently, it has been reported that TRPV1-expressing pancreatic sensory neurons control islet inflammation and insulin resistance in type I diabetes [143]. Ablation of these TRPV1 positive sensory afferents by capsaicin application prevents the onset of diabetes and associated pancreatitis in diabetes prone non obese diabetic mice [143]. 

### Taste and Food Intake

A TRPV1 receptor variant is attributed to be the mammalian amiloride-insensitive non-specific salt taste receptor [103]. This receptor is expressed in fungiform taste receptor cells and is a non-selective cation channel, activated by vanilloids, temperatures >38˚C, inhibited by capsazepine and is absent in TRPV1 knock-out mice [103]. Thus, developing specific salt taste suppressors or enhancers may prove beneficial in managing cardiovascular complications, such as hypertension. The activation of TRPV1 in the nodose ganglia by the endogenous feeding and body weight regulating lipid, oleoylethanolamide [3], resulted in visceral pain and reduced food intake in only wild type but not in TRPV1 knock-out mice [196]. Craving for spicy food may be as a result of capsaicin effect on the satiety centers [3]. It is interesting to note that the TRP channels that are purported to be involved in pain are also expressed in gustatory neurons and carry specific taste sensations. For example, TRPV1 carries the pungent taste associated with capsaicin and vinegar, whereas TRPA1 carries the taste associated with mustard, horse radish and wasabi. 

### Hearing

Capsaicin-mediated activation of the sensory nerves supplying the cochlea has been reported to increase cochlear blood flow [191,192,203]. This effect of capsaicin is attributed to the release of SP from the axons in the basilar artery, the anterior inferior cerebellar artery, the spiral modiolar artery, and the radiating arterioles of the cochlea [192]. TRPV1 immunoreactivity has been reported in the hair cells, organ of corti, vestibular and spiral ganglion cells of the cochlea [15, 203]. 5-LOX metabolites of AA activate TRPV1, TRPV1 and 5-LOX immunoreactivity has been observed in the neurons and satellite cells in adjacent sections of these ganglia [15]. TRPV1 activation in the cochlea is associated an increase in the threshold of auditory nerve compound action potentials and a reduced magnitude of cochlear microphonic and electrically-evoked otoacoustic emissions, suggesting an important modulatory role in the physiology of the inner ear [203]. In a recent study by Kitahara and colleagues [92], subcutaneous kanamycin administration in mice resulted in increased mRNA levels of TRPV1 as determined by the real-time PCR technique in the spiral and vestibular ganglia. The authors concluded that upregulated TRPV1 in these ganglia may promote neuronal survival and underlie kanamycin-induced dizziness and tinnitus as side effects. Furthermore, TRPV1 may also be implicated in hearing loss associated with inflammation in salicylates-induced tinnitus or in pathological conditions of the inner ear such as in Meniere’s disease or in migraines. Tinnitus can be equated to chronic pain conditions; both involve spontaneous neuronal firing and likely to be mediated *via* TRPV1.

### Hair

Expression of TRPV1 on the hair follicle (mainly in the outer root sheath and hair matrix) has been established by using diaminobenzidine immunoreactivity [25]. Using cultured human scalp hair follicles, capsaicin application was shown to inhibit hair shaft elongation and induce apoptosis, suppress proliferation and enhance premature hair follicle regression. Up-regulation of endogenous hair growth inhibitors and down-regulation of endogenous hair growth promoters were also observed in cultured outer root sheath keratinocytes upon TRPV1 activation [25]. Thus, the TRPV1 receptor is an important regulator of human hair growth and its expression in hair follicles and keratinocytes suggest a role beyond nociception.

### Functions of TRPV1

Noxious stimuli are transduced by peripheral nociceptors, which transmit information regarding tissue damage to pain-processing centers in the brain *via* the spinal cord [16]. TRPV1 in sensory nerve endings transduce various stimuli, in particular, inflammatory thermal pain as indicated by deletion of the TRPV1 gene [30, 42]. TRPV1 is involved in both afferent (sensation of pain) and efferent (neurotransmitter and neuropeptide release) functions, which are experienced with a burning sensation followed by vasodilation and sweating upon consumption of capsaicin in spicy foods. Thus, TRPV1 can mediate both inflammation and pain and this dual mode of action qualifies it to be an important player in various physiological and pathophysiological processes.

Important properties of the TRPV1 receptor include sensitization, desensitization and tachyphylaxis. Capsaicin, and certain noxious stimuli can induce a prolonged hypersensitive state and capsaicin-induced hyperalgesia is a well-characterized pain model. Following intradermal capsaicin injection in humans, pronounced hyperalgesia to heat and mechanical stimuli is experienced at the injection site, which later spreads to the surrounding tissue [172]. Desensitization is defined as a reduction in response to continued exposure to an agonist. Tachyphylaxis is defined as a reduction in response to repeated application of an agonist. Both desensitization and tachyphylaxis require extracellular Ca^2+^ [31], suggesting the involvement of Ca^2+^-dependent intracellular signaling mechanisms. Tachyphylaxis can be abolished by inhibitors of Ca^2+^-dependent phosphatases, suggesting the requirement for phosphorylation in channel activation [50]. Thus, Ca^2+^-mediated dephosphorylation may render the channel inactive leading to desensitization or tachyphylaxis. Prolonged TRPV1 activation by capsaicin may lead to its desensitization and this may account for the paradoxical use of capsaicin as an analgesic agent [171]. Interestingly, PKA-, but not PKC-mediated phosphorylation is able to reverse tachyphylaxis, suggesting distinct actions for these kinases [120, 121, 122]. Desensitization may result from Ca^2+^-induced modulation of TRPV1 sensitivity, and/or Ca^2+ ^toxicity; a large and sustained Ca^2+^ influx *via* TRPV1 has been linked to neurodegeneration of the peripheral nerve terminals. 

## ACTIVATION CHARACTERISTICS OF TRPV1

TRPV1 is a well-characterized channel, which transduces heat in the noxious temperature range (>42^o^C) and is critical for nociceptive and inflammatory thermal sensation [80]. It is a Ca^2+^ permeant polymodal receptor activated by protons, anandamide, lipoxygenase metabolites of arachidonic acid (AA), N-arachidonyl dopamine (NADA), capsaicin (an active ingredient in hot chili peppers) and RTX, an ultrapotent agonist obtained from the cactus, *Euphorbia resinifera* [29]. RTX combines structural features of phorbol esters (potent activators of PKC) and vanilloid compounds. It was thought that its ability to activate PKC might be responsible for its high potency, but the concentration required to activate PKC is much higher to account for this effect [63]. 

Cloning and mutational analysis of TRPV1 has improved our understanding of how these receptors are activated. The TRPV1 ion channel subunit has six transmembrane-spanning segments flanked by large intracellular, N- and C-terminal domains [31, 81, 94]. Therefore, an intracellular modulator or a readily membrane-permeable agent is required to activate/block the receptor. Binding of capsaicin and RTX to TRPV1 involves amino acid residues which have been shown to reside in N- and C-cytosolic and transmembrane domains of the channel [34, 57, 81, 82]. The binding site for vanilloid compounds has been reported to lie between transmembrane domains 2 and 4. Amino acids Y511 and S512 may play a key role in capsaicin binding [78]. Other studies have implicated the importance of residues on the cytoplasmic tail in capsaicin and proton binding [78]. Amino acids in transmembrane domain 6 (N676, M677 and L678) play a significant role in capsaicin- and proton-induced activation of TRPV1 [79]. Mutation of residues at D601, E610 or E648 to neutral or basic residues resulted in a loss of proton sensitivity. In contrast, mutation of E600 resulted in increased sensitivity to vanilloids at higher temperatures [79]. 

A potent endogenous activator of TRPV1 has not been identified. Anandamide, NADA and lipoxygenase metabolites of AA have been shown to activate the receptor weakly. However, in the phosphorylated state, agonist and temperature sensitivity is greatly increased, in that TRPV1 could be active at normal body temperature [130, 162, 168]. Therefore, the activation mechanism could include multiple stimuli integrating synergistically to induce a maximal response. 

## SENSITIZATION OF TRPV1 BY PHOSPHORYLATION

Activation of Ca^2+^ permeant nociceptive ion channels on the peripheral and central terminals of sensory neurons leads to the synthesis and/or release of a variety of proinflammatory agents and neuropeptides, like ATP, BK, PGs, CGRP, SP and vasoactive intestinal peptide (VIP) [105, 114]. Increases in intracellular Ca^2+^ initiate several second messenger pathways including the activation of phospholipase A2 (PLA_2_), phospholipase C (PLC) and Ca^2+^-dependent kinases, which can lead to the generation of AA and its metabolites, release of Ca^2+^ from intracellular stores and phosphorylation of nociceptive ion channels, respectively.

TRPV1 is important in inflammatory thermal hyperalgesia. Recent studies have suggested that various inflammatory mediators are able to sensitize TRPV1 to chemical and physical stimuli. Inflammation results in acidic conditions, which can activate TRPV1 directly. The inflammatory mediators activate their respective G-protein coupled receptors to initiate secondary messenger pathways resulting in the activation of either PKA [20, 44, 70, 121, 131, 141], PKC [19, 32, 39, 130, 138, 172, 183], MAPK [76, 204], extracellular Ca^2+^/CaM-dependent kinase II (CaMKII) [83, 149] or Src kinase [77], which phosphorylate TRPV1. 

Cyclic AMP-dependent protein kinase A (PKA) in primary afferents is important in mediating inflammatory hyperalgesia [144, 178, 179]. Activation of PKA by either using cAMP analogues or PGs results in hyperalgesia [178, 179]. PKA-mediated phosphorylation of TRPV1 potentiates capsaicin-mediated current and increase CGRP release in cultured DRG neurons [64, 70, 102]. Anandamide causes release of neuropeptides by activating both cannabinoid 1 (CB1) and TRPV1 receptors [5]. Activation of CB1 or CB2 receptors has been shown to increase or decrease adenylate cyclase levels, which will modulate the phosphorylation state of TRPV1. Activation of the CB1 receptors decreases Ca^2+^ and increases K^+^ conductance in the presynaptic terminals [33] that can interfere with the action of TRPV1 distributed at the central terminals of sensory neurons. Phosphorylation at S116 in the amino terminus of TRPV1 is vital in PKA-mediated regulation of TRPV1 desensitization [20]. Mutations of S116, T370, T144, and S502 are capable of reversing forskolin-induced potentiation of TRPV1 responses [20, 121, 122, 141]. 

Phosphorylation by PKC has been shown to sensitize TRPV1 [32, 138]. Various algesic agents like BK, ATP, trypsin and PGs are known to sensitize TRPV1 by activating PKC downstream of their G-protein coupled receptors in sensory neurons and in expressed systems. PKC-dependent phosphorylation of TRPV1 not only potentiates capsaicin- or proton-evoked responses but also reduces its temperature threshold for activation [39, 126, 168, 183]. Serine residues (S502 and S800) on TRPV1 have been identified by biochemical studies to be important in PKC-mediated effects [19, 130]. 

Phorbol esters can sensitize TRPV1 by phosphorylating the channel [32, 138, 193]. They have also been reported to activate the channel directly as suggested by the induction of capsazepine-sensitive inward currents in HEK cells transiently transfected with hTRPV1 [193]. PKC-mediated phos-phorylation of TRPV1 can reduce its activation threshold by partially activating the receptor such that lower concentrations of the agonist can fully activate the receptor [4, 138, 193]. In addition, PKC alters the time- and voltage-dependent properties of TRPV1. PKC-mediated phosphorylation of TRPV1 in DRG neuronal cultures has been shown to produce faster activation and slower deactivation of the channel [4]. Phosphorylation not only sensitizes the receptor and augments its response, but also promotes translocation of TRPV1 from the cytosol to the plasma membrane [124, 167, 190].

PKCε, and PKCα, calcium-independent and calcium-dependent isoforms of PKC, respectively, have been shown to phosphorylate TRPV1 [29, 32, 138, 193]. PKC plays a pivotal role in pain signaling, and the PKCε gene disruption in mice dramatically reduces thermal and inflammatory pain [89]. In mice lacking PKCγ, acute pain was preserved, but the neuropathic pain was reduced [107]. 

Ca^2+^ calmodulin-dependent protein kinase II (CaMKII) has been shown to modulate TRPV1 function and capsaicin binding [83, 149]. In contrast, calcineurin-mediated dephosphorylation causes TRPV1 desensitization [50]. Mutants of CaMKII phosphorylation sites on TRPV1 (S502 and T704) failed to elicit currents in response to application of capsaicin or RTX [83]. Thus, CaMKII and calcineurin control the subtle balance between TRPV1 phosphorylation/dephosphory-lation and its activation, deactivation and desensitization characteristics. 

TRPV1 is activated by membrane-derived lipids like anandamide, oleoylethanolamide and some lipoxygenase products of arachidonic acid [3, 73, 205]. Phosphainositol 3, 4 bisphosphate (PIP2) has been suggested to tonically inhibit TRPV1 by its constitutive association with the channel. Activation of PLC by metabotropic receptors (BK2, TrkA and histamine) hydrolyzes PIP_2_ into diacylglycerol and inositol (1, 4, 5) trisphosphate, which relieves its tonic inhibition of the channel [35]. PIP_2_ regulates TRPV1 activity by interacting with amino acids between 777–820, which contains many positively charged residues [139]. This region also includes S800, the PKC phosphorylation site and overlaps with the 35 amino acid segment necessary for CaM binding. This region might thus be very important for the activation of TRPV1. BK and histamine are coupled to PLA_2_, activation of which, in turn, can produce AA and its metabolites leading to the modulation of TRPV1 [160].

## TRPV1 AGONISTS

The possibility of using TRPV1 receptor agonists to alleviate pain is an interesting concept. Activation of TRPV1 can induce persistent depolarization of the nerve terminals causing a decrease in their ability to generate and propagate action potentials. On the other hand, TRPV1 activation causes an influx of Ca^2+^ that in the long-term can cause nerve terminal degeneration. Ablation of TRPV1 containing neurons by intraperitoneal injection of capsaicin or RTX has been demonstrated [127, 133, 200]. Furthermore, high concentration of capsaicin has been used to alleviate pain associated with herpes zoster, AIDS neuropathy, diabetic peripheral neuropathy, etc [80, 172]. In this strategy to avoid the pain caused by capsaicin, a prior application of lidocaine is warranted. Local application of 0.075% capsaicin ointment has been reported to have moderate to poor efficacy following eight weeks of application in neuropathic or musculoskeletal pain [113]. In itch-causing pruritis, capsaicin 0.006% has been found to be useful [104].

Intrathecal administration of capsaicin has been shown to cause a long-lasting loss of thermal sensitivity without affecting mechanical sensitivity [200]. RTX is a potent agonist of TRPV1, which exhibits unique properties that can be exploited to treat chronic pain conditions [140]. The characteristics of RTX include the following: 1. irreversible nature of activation enables the use of low concentrations (well below the toxic levels); 2. slow and sustained depolarization results in a gradual inactivation of voltage-gated Na^+^ channels causing a depolarization block, preventing action potential generation in the short-term and Ca^2+^-induced nerve terminal death in the long-term [140]. Since the nerve terminals have the ability to regenerate, long-term toxicity may not be a major concern. Intrathecal administration of RTX causes a loss of thermal sensitivity [175]. Idalora and colleagues [27] have used intrathecal administration of RTX to alleviate pain associated with osteosarcoma in dogs. We have found that selective ablation of central terminals of DRG neurons by intrathecal administration of RTX can achieve reduction of nociceptive neurotransmission. RTX in very low (nanomolar) concentrations can activate TRPV1 slowly, maximally and irreversibly leading to depolarization beyond the threshold without generating action potentials. RTX-induced sustained depolarization leads to the loss of generation of action potentials in the peripheral and/or central TRPV1 containing nociceptor nerve terminals in the short-term and ablation of these nerve terminals in the long-term. This has the potential to alleviate chronic pain conditions (S.Y. and L.S.P., unpublished results). The prevention of permanent loss of the DRG is therapeutically favorable and offers a unique approach to treat chronic pain conditions. The selectivity of RTX action on TRPV1-containing nociceptive nerve terminals may offer an advantage of a higher therapeutic index compared to other clinically available agents (toxins), such as, botulinum toxin and ω-conopeptide, which act on synaptic vesicles and on Ca^2+ ^channels, respectively. 

## TRPV1 ANTAGONISTS

For decades the only antagonist available to inhibit capsaicin-mediated responses was the organo-metallic dye ruthenium red [53]. Ruthenium red is a non-competitive antagonist of TRPV1; however, based on recent studies it can be classified as a general inhibitor of TRPV channels [60, 164]. The mechanism of action is proposed to be by blocking the channel pore. Moreover, it has also been demonstrated to block other channels, in particular Ca^2+^ channels [53]. The well-studied TRPV1 antagonist is the synthetic vanilloid analogue, capsazepine [18, 187]. Capsazepine is a competitive antagonist of TRPV1, which is able to block both *in vivo* and *in vitro* effects of capsaicin and exhibits a relatively high selectivity for TRPV1 as compared to other TRPV channels. However, capsazepine is far from being an ideal TRPV1 antagonist as it has many disadvantages. First, it is a very weak antagonist with IC_50_ values of 0.2-4 μM [2, 18, 31, 101, 188, 199]. Second, it demonstrates species-dependent difference in inhibiting TRPV1. Capsazepine inhibits acid-induced activation of human TRPV1 (hTRPV1), but has no inhibitory effect on acid-induced activation of rat TRPV1 (rTRPV1) [190]; however, it can inhibit heat-mediated responses in both hTRPV1 and rTRPV1. Third, at concentrations needed to inhibit TRPV1 (~10 μM), it has been demonstrated to block amongst many receptors and channels, voltage-gated calcium channels [51] and acetylcholine receptors [101]. Furthermore, the sarcoplasmic reticulum ATPase (SERCA) inhibitor, thapsigargin, was shown to inhibit the capsaicin-induced calcium influx with an IC_50_ of 6.4 ± 1.9 μM as well as prevented [^3^H] RTX binding (IC_50_ = 4 ± 1.3 μM) in CHO cells expressing TRPV1 [186].

Iodination of RTX at the 5΄ position of the vanilloid moiety resulted in one of the most potent TRPV1 antagonist iodo-resiniferatoxin (iodo-RTX) [196]. Iodo-RTX has been reported to inhibit capsaicin-induced currents in *Xenopus laevis* oocytes expressing TRPV1 with an IC_50_ value of 3.9 nM. Intrathecal administration inhibited capsaicin-elicited pain responses with ED_50_ of 16 ng/mouse [196]. Furthermore, iodo-RTX has been demonstrated to inhibit both heat- and proton-evoked responses in sensory neurons as well as cells expressing the recombinant TRPV1 [145]. However, in an interesting study by Shimizu and colleagues [159], systemic administration of iodo-RTX in mice resulted in hypothermia, as these effects were not seen in either TRPV1 knock-out mice or RTX-pretreated animals. Iodo-RTX was also shown to enhance intracellular calcium levels and induced a small inward current in HEK293 cells stably transfected with TRPV1. A possible suggested explanation for the partial agonistic action of iodo-RTX is that it is metabolized (deiodination) to RTX *in vivo*. The controversy over the antagonistic *vs*. partial agonistic action of iodo-RTX has resulted in a loss of enthusiasm over this compound.

Since TRPV1 has become an undisputed target for a variety of painful conditions, a quest for synthesizing potent yet selective TRPV1 antagonists is unabated. A non-vanilloid TRPV1-antagonist N-(4-tertiarybutylphenyl)-4-(3-chloropyridin-2-yl)tetrahydropyrazine-1(2H)-carbox-amide (BCTC) has been synthesized. It inhibits capsaicin- and acid-induced responses in rat TRPV1 with an IC_50_ value in the range of 6-35 nM; unlike capsazepine, however, BCTC does block acid-mediated activation of rat TRPV1 [190]. Furthermore, orally administered BCTC (3-30 mg/kg) has also been demonstrated to inhibit both mechanical and thermal hyperalgesia associated with complete Freund’s adjuvant-induced inflammation and nerve injury [137] without affecting normal/acute nociception. BCTC is lipophilic and crosses the blood-brain barrier and may carry the risk of inducing central nervous system side effects. However, in its analgesic concentrations, BCTC has not been documented to produce any locomotor impairment usually associated with centrally acting analgesics.

The Glaxo Smith Kline compound SB-366791 is a competitive inhibitor of TRPV1 that inhibits capsaicin, acid and noxious heat-mediated activation of the receptor. It is superior to capsazepine as it blocks acid-mediated activation of rTRPV1. It has demonstrated a high selectivity in 47 different binding and electrophysiological assays [61]. Furthermore, SB-366791 does not inhibit VGCC or hyperpolarization-activated current (I_h_) in cultured rodent sensory neurons. In a recent study, spontaneous and miniature excitatory synaptic currents (sEPSC and mEPSC), recorded from the substantia gelatinosa neurons of the spinal cords obtained from CFA-injected animals, were decreased in the presence of SB-366791 [96].

Abbott laboratories have in the recent years synthesized a number of promising TRPV1 antagonists. The A-425619 [1-Isoquinolin-5-yl-3-(4-trifluoromethyl-benzyl)-urea] is a com-petitive, potent (IC_50_= 5-9 nM) and a highly selective antagonist for the capsaicin receptor [173]. It inhibits increases in intracellular calcium influx evoked by capsaicin, NADA, anandamide or pH in HEK cells transfected with hTRPV1 as well as capsaicin-induced inward currents in cultured rat DRG neurons. Furthermore, in experiments, where TRPV1 receptors expressed in HEK cells were sensitized by a PKC activator, PDBu or low pH 6.0, capsaicin-mediated intracellular influx was inhibited by A-425619 at much lower concentrations (pA_2_ = 0.8 ± 0.3 and 4.3 ± 1.9 nM, respectively). Recently, a detailed study highlights the properties of two of their leading contenders for antagonizing TRPV1 receptor that are orally active. A-784168 (1-[3-(trifluoromethyl)pyri-dine-2-yl]-N-[4-(trifluoromethylsulfonyl)phenyl]-1,2,3,6-tet-rahydropyridine-4-carboxamide) and A-795614 (N-1H-indazol-4-yl-N’- [(1R)-5-piperidin-1-yl-2,3-dihydro-1H-inden-1-yl]urea) have comparable efficacy in in-vitro assays (IC_50_= 25 ± 2 and 14 ± 1 nM, respectively, in inhibiting capsaicin-mediated responses), but have significant differences in their ability to permeate the CNS [38]. Interestingly, in models representing chronic pain conditions, including CFA- and capsaicin-induced mechanical allodynia and osteoarthritis, both agents demonstrated equal efficacy when administered intrathecally; however, when administered orally, A-784168, which crosses the blood-brain barrier, was found to be superior than A-795614 [38]. This study clearly demonstrates that drugs which cross the blood-brain barrier are more efficient in antagonizing TRPV1 *in vivo*. However, it could also be argued that such highly lipophilic compounds may be associated with a greater CNS side effect profile, because of TRPV1 distribution in the brain. 

Amgen has developed a piperazinylpyrimidine analog (AMG517) as a potent TRPV1 antagonist, which is in the phase 1 clinical trials. Amgen has also developed other potent compounds (AMG0347, AMG8163, AMG9810), IC_50_ values to antagonize capsaicin responses range between 0.5 to 86 nM [25]. Neurogen/Merck has launched NDG-8243/MK-2295 in Phase II clinical trials, the structure of which is unpublished/unreported. The latest compound to enter clinical trials is GRC-6211 from Glenmark pharmaceuticals. Other TRPV1 antagonists include JNJ-17203212 (Johnson and Johnson) has been shown to be effective in bone cancer pain and JYL1421 (Schwarz Pharma), which blocks capsaicin induced responses with an IC_50_ of 9.2 µM [173].

The NGF antibody is being evaluated for the treatment of chronic pain. NGF has been shown to sensitize TRPV1 by activating PLC and hydrolyzes PIP2, which tonically inhibits the channel [35, 139], or by activating PKC, PI3K [26, 35, 161, 167] and ERK [204]. Although the precise mechanism of action is debatable, it is known that NGF sensitizes TRPV1 and increases its expression and function. Therefore, blocking NGF action could down regulate TRPV1 expression and function. 

## CONCERNS OF TRPV1 ANTAGONISTS

TRPV1 antagonists hold a great promise in alleviating multiple modalities of pain. However, it must be borne in mind that other physiological functions that are mediated by TRPV1 channels may be affected. Of concern are the maintenance of body temperature, TRPV1-mediated SP and CGRP release and their subsequent effect on the cardiovascular functions and acute nociception. Particular efforts should be focused on changes in body temperature. In some studies, the body temperature has been shown to rise as much as 3^o^C. Cardiovascular consequences range from the inability to feel pain during myocardial ischemia to decreased TRPV1-mediated CGRP release that can compromise coronary circulation. CGRP is also critical for the integrity of microvascular circulation and lack of which could aggravate diabetic peripheral neuropathy and peripheral vascular disease. Gastrointestinal consequences could occur as a result of a decreased release of neuroactive substances, as well as by modulation of vagal nerve circuitry. The preferred route of oral administration of TRPV1 antagonists could have an immediate impact on the gastrointestinal functions. The main disadvantage with the existing nonselective COX inhibitors is that they increase gastric acid section that may lead to life threatening bleeding due to decreased PG synthesis. However, studies have suggested that capsaicin is capable of releasing HCO_3_^-^ as efficiently as acid challenge. Therefore, blocking TRPV1 may decrease the release of HCO_3_^-^, which is essential to neutralize the acid. Recent studies show that the TRPV1 knock-out animals have deficiencies in fear conditioning and memory formation, which are serious issues to consider. RTX is in clinical trials for the treatment of urinary bladder hyperreflexia. Therefore, it should be evaluated how the block of TRPV1 could affect normal bladder functions. Furthermore, blocking TRPV1 has been shown to increase its own expression, thereby raising the possibility of rebound effects. Given the observations that TRPV1 knock-out mice have normal life span without serious abnormalities, suggest that the block of TRPV1 may not induce serious adverse effects. However, it has to be investigated how these animals react upon insult or in pathological conditions.

## CONCLUDING REMARKS

Although, TRPV1 is considered to be a potential target for developing drugs to treat different modalities of pain, extensive basic science research and clinical trials have to be undertaken to uncover possible adverse effects. This task is further complicated by the advent of TRPV1 distribution in novel areas mediating unexpected functions. In particular, antagonizing the receptor may lead to cardiovascular complications as a result of decreased vasoactive peptide release. Sustained stimulation can also lead to Ca^2+^-dependent nerve terminal degeneration. Therefore, the consequence could be as a result of both agonistic and antagonistic actions. Finally, there is no drug that has been invented without adverse effects; therefore, the balance between the beneficial and the adverse effects should be cautiously and pragmatically considered. Using agents to selectively target nerve terminals at the periphery or at the level of the spinal cord may avoid some of the systemic adverse effects. In any case, TRPV1 is a novel target for next generation analgesics, and if proven to be successful in clinical trials, its agonists and antagonists may be added to the therapeutic armamentarium.
